# Prevalence and characteristics of hepatitis B and D virus infections among HIV-positive individuals in Southwestern Nigeria

**DOI:** 10.1186/s12985-021-01493-4

**Published:** 2021-01-15

**Authors:** Oluyinka Oladele Opaleye, Olusola Anuoluwapo Akanbi, Folakemi Abiodun Osundare, Bo Wang, Olufisayo Adesina, Adeolu Sunday Oluremi, Sola Thomas Sunday, Abiodun Akeem Akindele, Patrycja Klink, C. Thomas Bock

**Affiliations:** 1 Department of Medical Microbiology and Parasitology, Ladoke Akintola University of Technology, Ogbomoso, Oyo State Nigeria; 2 Division of Viral Gastroenteritis and Hepatitis Pathogens and Enteroviruses, Department of Infectious Diseases, Robert Koch Institute, Berlin, Germany; 3 Department of Microbiology, Obafemi Awolowo University, Ile Ife, Osun State Nigeria; 4 Institute of Tropical Medicine, University of Tuebingen, Tuebingen, Germany

**Keywords:** HBV, HDV, HIV, Co-infection, Drug resistance, ART, Nigeria

## Abstract

**Background:**

Coinfections of HIV-positive individuals with Hepatitis B and D virus (HBV and HDV) are common and can be associated with rapid liver damage. Several antiretroviral drugs for HIV exhibit anti-HBV effect; however, the selection of HBV drug resistance mutations (DRMs) in individuals under HIV antiretroviral therapy (ART) has been reported but rarely in Nigeria. In this study the HBV/HDV prevalence and HBV DRMs in HIV-positive individuals in Southwestern Nigeria were assessed.

**Methods:**

Plasma samples collected from 310 HIV-positive individuals including 295 ART-experienced and 15 ART-naïve persons attending the HIV clinic in three south-western states of Nigeria between June 2017 and August 2017 were analysed by ELISA for HBsAg and anti-HDV. The presence of HDV RNA and HBV DNA was analysed by (RT)-PCR followed by sequencing and phylogenetic analyses for genotyping. The HBV reverse transcription (RT) region was amplified and sequenced for the analysis of drug resistance mutations.

**Results:**

Overall, 16.1% (n = 50/310) of the HIV-positive individuals were positive for HBsAg, most of which were ART-experienced (94.0%; n = 47/50). From the 50 HBsAg-positive samples, 72.0% (n = 36/50) were positive for HBV DNA and 16.0% (n = 8/50) had detectable HDV RNA while 5.6% (n = 2/36) of the HBV-DNA positive samples had anti-HDV total antibodies. Sequences were available for 31/36 of the HBV DNA-positive and 3/8 HDV RNA-positive samples. HBV DNA-positive samples were characterised as HBV genotype E infections exclusively, while HDV genotype 1 was detected in the HDV RNA-positive samples. HBV DRMs V173L, L180M, S202I and M204V/I, which are associated with lamivudine resistance, were detected in 32.2% (n = 10/31) of the HBV DNA-positive samples. Most of these mutations (90.0%; n = 9/10) were present in the ART-experienced cohort.

**Conclusions:**

This study indicates that HBV/HDV coinfections are common in HIV-positive individuals under ART in Nigeria. Furthermore, a high proportion of HBV DRMs which potentially compromise future treatment options were detected, underscoring the need for HBV screening prior to starting ART. Further studies should be performed to monitor a possible increase in the spread of HDV among populations at risk of HIV and HBV infections.

## Background

Due to shared routes of transmission, co-infection of HIV with hepatitis viruses is common worldwide and it is estimated that 5–20% of the approximately 37 million people living with HIV are co-infected with HBV [1]. In Africa (Nigeria inclusive), HBV is hyper-endemic and HBV/HIV co-infections are highly prevalent [2]. Compared to HBV or HIV mono-infected individuals, co-infected individuals have a higher risk of impaired immunological recovery and hepatotoxicity during antiretroviral treatment (ART) and a faster rate of progression to cirrhosis and hepatocellular carcinoma [3, 4].

The prevalence of HDV is about 5% among HBV carriers however, rates between 3 and 30% have been reported in West Africa [5–7]. HDV co-infection increases the risk for hepatitis flares and chronic hepatic complications especially in individuals with HBV/HIV co-infection [8]. However, the impact of HDV infection on HIV/HBV co-infected individuals is not well documented, especially in Nigeria where HDV is not routinely diagnosed.

Currently, six nucleoside analogues have been approved for the treatment of chronic HBV infection, all of which are inhibitors of the reverse transcriptase (RT) of HBV [9] and some of them (3TC and TDF/TAF) are also part of the first line regimen used for HIV ART [10]. Administration of these drugs in HIV-positive patients without knowledge of their HBV status can lead to the emergence of HBV drug resistance mutations (DRMs) and potentially limiting the treatment options available for the treatment of HBV [11]. Therefore, the World Health Organisation (WHO) [12] has recommended that all HIV-infected patients should be tested for the presence of HBV prior to initiation of therapy [12], but this is rarely done in Nigeria and some parts of sub-Saharan Africa due to high costs or partly due to neglect of the disease and concentration on HIV treatment [13, 14].

The HBV status of most HIV-infected individuals remains unknown, and first line ART regimen for HIV contains zidovudine (AZT), lamivudine (3TC) and nevirapine (NVP) in Nigeria (https://www.who.int/hiv/pub/guidelines/nigeria_art.pdf). As a result of the low genetic barrier to resistance of 3TC, [15] the risk for the emergence of drug resistant HBV strains is increased. Moreover, studies have shown that HBV mutations can occur even in the absence of 3TC therapy in drug-naïve HBV carriers, thereby contributing to the rapid development of drug resistance [16].

Thus, knowledge of HBV and HDV status among HIV-infected patients is important for adequate clinical monitoring and selection of ART regimen. This study was carried out to investigate the prevalence and genotype distribution of HBV and HDV in HIV-positive individuals in Southwestern Nigeria. Furthermore, the presence of HBV DRMs in ART-experienced and naïve HIV-infected individuals was analysed.

## Methods

### Study population

Plasma samples were randomly collected from 310 HIV-positive individuals including 295 ART-experienced and 15 ART-naive HIV-positive persons. The samples were collected from the HIV clinics of Adeoyo hospital, Ibadan, Oyo state (82 samples) and LAUTECH teaching hospital, Osogbo, Osun state (100 samples) and Offa, Kwara state (128 samples) both located in the southwestern region of Nigeria. Samples were collected between June and August 2017. Informed consent of each participant was obtained before sample collection and the study was approved by the Ethical review board of LAUTECH Teaching Hospital, Osogbo, Nigeria. Samples were stored at − 80 °C until testing and all laboratory analyses were carried out at the Robert Koch Institute, Berlin, Germany.

### HBV and HDV serology

Qualitative detection of HBsAg was performed using the Wantai HBsAg ELISA (Wantai Hepatitis B virus Diagnostics, China). Anti-HDV antibodies were analysed using the ETI-AB-DELTAK-2 enzyme immune-assay (anti-HD; DiaSorin Limited, Germany) for the qualitative determination of total antibodies to hepatitis delta antigen. All tests were performed following the manufacturer’s instructions.

### Nucleic acid extraction and PCR amplification

Total viral nucleic acids were extracted from 140 μl of plasma using the QIAamp MinElute Virus Spin Kit (Qiagen, Hilden, Germany) using the QIACUBE automated system (Qiagen, Hilden, Germany). Nucleic acids were eluted in 60 µl of nuclease-free elution buffer and stored at − 80 °C until use. For HBV genotyping, the partial HBV S-gene was amplified using nested PCR as previously described [17]. HBV DRMs were detected by the amplification of a partial coding region of the HBV RT-region designated for antiviral resistance using nested PCR followed by population-based Sanger sequencing.

HDV was genotyped by HDV-specific nested RT-PCR using primers designed for the amplification of a highly conserved region of the HDV genome (LHDAg region, nucleotide 888 to 1122) and sequencing as previously described [6, 18]. All PCR products were analysed on 1.5% Agarose gels.

### HBV quantitative polymerase chain reaction

The HBV viral load was determined by quantitative real-time PCR (qPCR) using the Roche Lightcycler 480 (Roche, Mannheim, Germany) as described previously using primers in the region of overlap between the S-gene and P-gene resulting in a product of approximately 89 bp in size [17]. Absolute quantification of HBV genomes was carried out and expressed in copies per ml of plasma. HBV plasmid DNA was used to generate a standard curve following a serial 10-fold dilution. Our quantitative HBV-specific PCR assays were routinely standardized using the WHO standard (NIBSC code: 97/750). In addition, the detection of HBV DNA by real-time PCR in our laboratories was successfully validated by Quality Control for Molecular Diagnostics (QMCD, which is an independent International External Quality Assessment (EQA) / Proficiency Testing (PT) organization) round robin tests.

### Sequencing and phylogenetic analyses

Prior to sequencing, PCR amplicons were purified using ExoSAP-IT (Thermo Fischer Scientific, USA). Cycle-sequencing was performed on an ABI 3500 automated genetic analyser (Applied Biosystems Inc., Foster city, California, USA) with 1.0 μl of the BigDye® Terminator v3.1 Ready Reaction Mix (Applied Biosystems Inc., Foster city, California, USA) and 0.5 μl of each primer as described previously [6, 17]. Consensus sequences were generated by alignment of both sequenced strands with forward and reverse primers and subsequent manual edition to evaluate ambiguities using Geneious 10.0.5 (Biomatters Limited, Auckland, New Zealand). A Neighbor-Joining tree method with the maximum likelihood approach was constructed from consensus sequences using the MEGA 7 software version 7.0.26 [19]. For HBV phylogeny, representative reference sequences representing the HBV genotypes A-H and for HDV, reference sequences for genotypes 1–8 were randomly selected and retrieved from GenBank. Mutation analysis and prediction was done using BioEdit version 7.2.5. (http://www.mbio.ncsu.edu/BioEdit/bioedit.html), the geno2pheno [hbv] 2.0 online tool (https://hbv.geno2pheno.org/) and the HBVSeq tool of the Stanford HBV reverse transcriptase sequence variant database (HBVrtDB) [20]; https://hivdb.stanford.edu/HBV/HBVseq/development/HBVseq.html) online tool.

### Statistical analysis

Statistical analyses were done by Fischer’s exact tests with SPSS version 20.0.1 for Windows. A *p* value < 0.05 was considered to be statistically significant.

## Results

### Characteristics of the study population

The study population included 310 HIV-positive individuals with a median age of 40 years (range 4–73 years) and a median CD4 count of 459 cells/ml (range 6–2028 cells/ml). Of these, 63.2% (n = 196/310) were females while 36.8% (n = 114/310) were males. Most of the study participants (95%; n = 295/310) were on HIV ART (median age = 40 years; range 8–60 years; median CD4 count 462 cells/ml; range 2–2028) with combipack (a fixed-dose formulation of AZT + 3TC + NVP). Only5% (n = 15/310) of the study participants were ART-naïve (median age = 40 years; range 8–73; median CD4 count = 392 cells/ml; range 219–960).

### Prevalence of HBV and HDV infections in HIV-positive individuals

In total, 16.1% (50/310) of the HIV-positive individuals were positive for HBsAg with a prevalence of 15.9% (n = 47/295) in the ART-experienced cohort and 20% (n = 3/15) in the ART-naïve cohort (Table 1). The majority of individuals tested positive for HBsAg (94%; n = 47/50) were ART-experienced.

**Table 1:** Characteristics of study participants

	HIV (ART-experienced)	HIV(ART-naive)	Total
Patient number	295	15	310
Median age (range) years	40 (4–60)	40 (8–73)	40 (4–73)
Gender			
Female, n (%)	184 (62.4)	12 (80)	196 (63)
Male, n (%)	111 (37.6)	3 (20)	114 (37)
Median CD4 cell count (range) cells/ml	462 (6–2028)	392 (219–960)	459 (6–2028)
HBsAg +ve, n (%)	47 (15.9)	3 (20)	50 (16.1)
HBV DNA, n (%)	35 (11.9)	1 (6)	36 (11.6)
Median HBV viral load (copies/ml)	1.44 × 10^5^	4.36 × 10^6^	1.39 × 10^5^
Anti-HDV, n (%)	2 (0.7)	0 (0)	2 (0.6)
HDV-RNA, n (%)	7 (2.4)	1 (6)	8 (2.6)

+ve, positive

HBV DNA was detected in 72% (n = 36/50) of the HIV/HBsAg-positive individuals, with 97% (n = 35/36) being ART-experienced. The median HBV viral load was slightly lower in individuals being ART-experienced in comparison to ART-naïve individuals (1.44 × 10^5^ vs. 4.36 × 10^6^ copies/ml) as well as the median CD4 counts (497.85 cells/ml vs. 243 cells/ml) in the ART-experienced and the ART-naïve individuals, respectively (Table 1).

Among HBV DNA-positive samples, anti-HDV antibodies were detected in 5.6% (n = 2/36), all of which were HDV RNA-negative. 16.0% (n = 8/50) of the HBsAg-positive samples were positive for HDV RNA but negative for anti-HDV.

### HBV and HDV genotyping

**Fig. 1:**
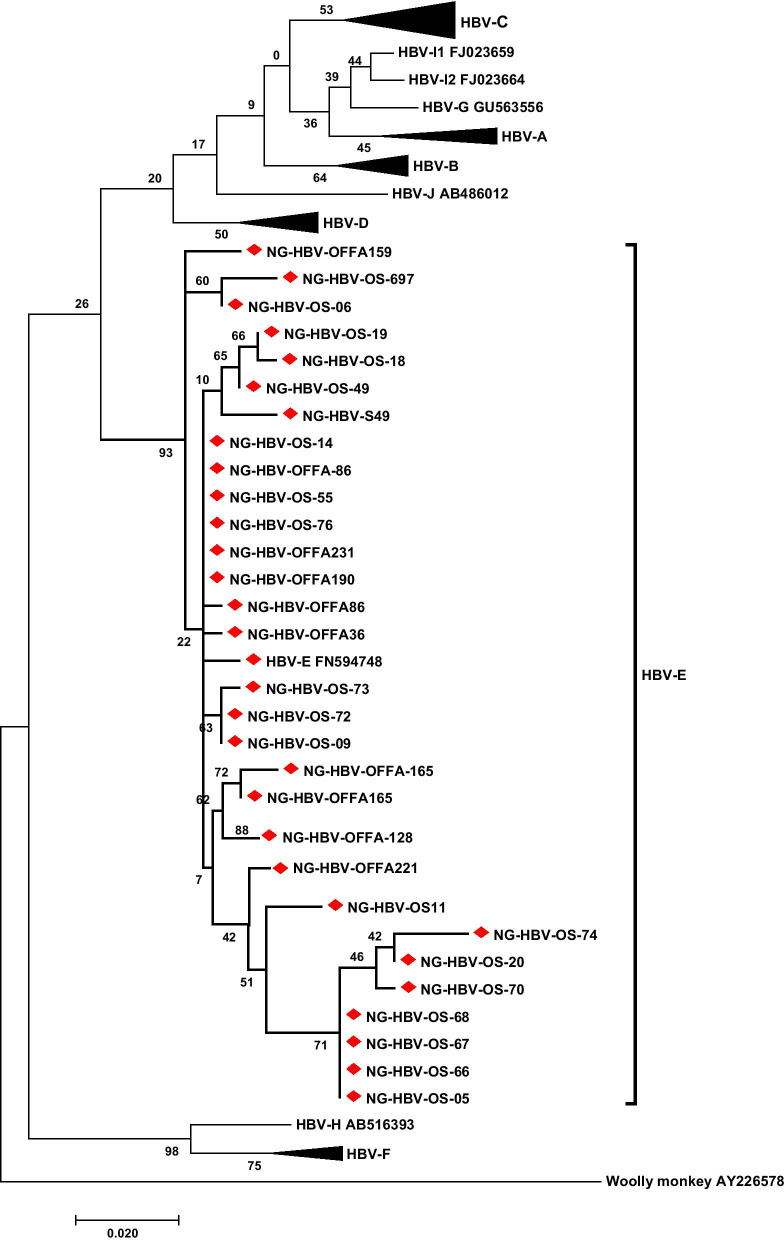
Phylogenetic analysis of the HBV isolates circulating among the HIV/HBV co-infected population in this study. The evolutionary history was inferred by using the Maximum Likelihood method based on the Tamura-Nei model. The tree with the highest log likelihood (-2314.93) is shown. The percentage of trees in which the associated taxa clustered together is shown next to the branches. The isolates (denoted by NG-HBV) were analysed with respect to reference sequences retrieved from GenBank which are designated by their respective accession numbers along with their HBV genotypes/subgenotypes. HBV sequences of the isolates from this study are available at NCBI GenBank database (Acc. No.:MK239481–MK239512)

To determine the HBV genotypes in the study cohort, the HBV isolates from this study were genotyped by sequencing and phylogenetic analyses of a partial region of the S and/or P genes. Of the 36 HBV DNA-positive samples 31 (86.1%) were successfully genotyped and all belonged to HBV genotype E (Fig. 1).

**Fig. 2:**
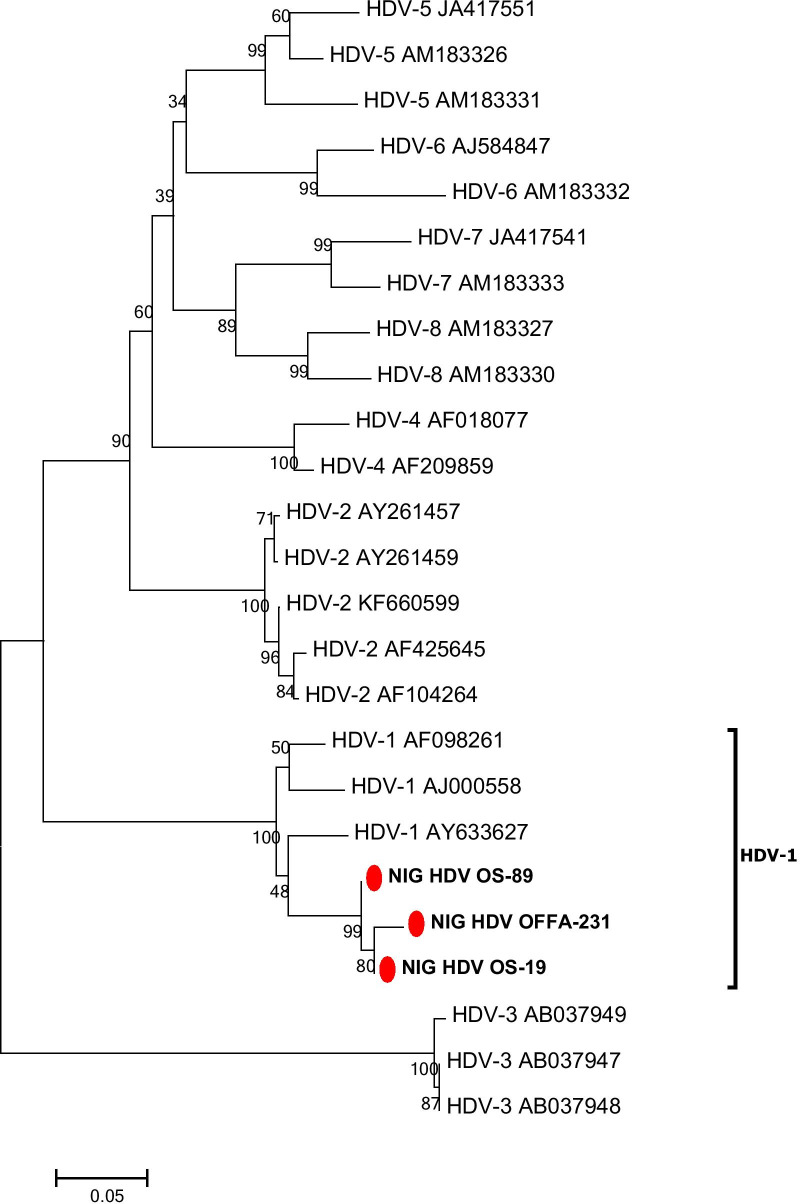
Phylogenetic analysis inferred from distance analysis (Kimura 2 parameters model) using neighbour-joining bootstrap 1000 replicate reconstruction from HDV sequences of the Nigerian HDV isolates in this study (highlighted in boldface and designated as NIG/HDV and numbers) and the corresponding region of reference sequences showing that the Nigerian HDV isolates clustered in the HDV genotype 1 branch. The Nigerian HDV sequences were compared to HDV reference sequences gathering the 8 HDV genotypes (HDV1 to HDV8). The numbers at the nodes indicate bootstrapping values. The bar represents nucleotide substitutions per position. HDV sequences of the patient isolates are available at NCBI GenBank database (Acc. No.:MK239513–MK239515)

Three of eight (37.5%) HDV RNA-positive samples could be successfully sequenced and showed HDV genotype 1 (Fig. 2).

### HBV drug resistance mutations

**Fig. 3:**
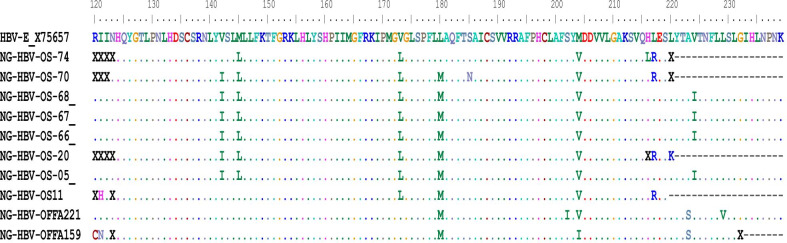
Representative HBV-amino acid (AA) sequences of the HIV/HBV coinfected individuals with DRMs aligned with HBV reference sequence X75657. HBV-AA sequences spanning the P-region of HBV from aa 120 to aa 240 showing patient-specific HBV isolates

HBV DRMs were detected in 32.3% (n = 10/31) of the successfully sequenced HBV DNA-positive samples (Table 2). Amino acid substitutions were detected in the following frequency: rtM204V/I (9/31; 29.0%), rtL180M (8/31; 25.8%), rtV173L (7/31; 22.5%). All of these substitutions are associated with a reduced susceptibility to lamivudine. In total, DRMs were detected in 10 individuals, of which 9 (90%) were ART-experienced and were treated with a fixed combination of zidovudine, lamivudine, and nevirapine (AZT/3TC/NVP). No single mutation was detected while all amino acid substitutions were detected in combinations as rtL180M + rtV173L + rtM204V (n = 7/10), rtV173L + rtM204V (1/10), rtL180M + rtM204I (1/10), and rtL180M + rtM204V + rtS202I (1/10). One of the individuals showing the DRM combination rtM204V/I + rtL180M + rtS202I was an ART-naive HIV/HBV/HDV-positive patient (Table 2; Fig. 3).

**Table 2:** Characteristics of individuals with HBV drug resistance mutations

ID	Age (years)	Gender (F/M)	ART regimen	HBV viral load (copies/ml)	CD4 count (cells/ml)	HDV-RNA (+/−)	HDV genotype	HBV-mutations P-region
OS05	33	M	Combipack	1.34 × 10^7^	700	+	**−**	L180M, V173L, M204V
OS11	44	M	Combipack	3.36 × 10^3^	800	**−**	**−**	L180M, V173L, M204V
OS20	26	F	Combipack	9.87 × 10^2^	740	**−**	**−**	L180M, V173L, M204V
OS66	41	F	Combipack	2.26 × 10^6^	150	**−**	**−**	L180M, V173L, M204V
OS67	23	F	Combipack	1.46 × 10^7^	625	**−**	**−**	L180M, V173L, M204V
OS68	41	M	Combipack	1.10 × 10^5^	640	**−**	**−**	L180M, V173L, M204V
OS70	28	F	Combipack	2.11 × 10^2^	800	+	**−**	L180M, V173L, M204V
OS74	27	M	Combipack	2.62 × 10^3^	385	+	**−**	V173L, M204V
OFFA159	49	F	Combipack	5.75 × 10^6^	1283	**−**	**−**	L180M, M204I
OFFA221	37	F	Naïve	4.36 × 10^6^	243	+	1	L180M, M204V, S202I

Combipack, zidovudine + lamivudine + nevirapine (AZT/3TC/NVP); P-region, Hbv polymerase region (reverse transcriptase)

## Discussion

Co-infection of HBV and HDV with HIV is still a major public health problem in Sub-Saharan Africa due to the high endemicity of these virus infections and the more severe clinical outcomes of coinfections in comparison to the mono-infections [4, 6, 21]. A vaccination programme against HBV (effective also for HDV) has been introduced in Nigeria since 2004; however, the prevalence of HBV remains high (10–15% [6, 22] compared to a prevalence of 20–25% before the advent of the vaccination [23]). The prevalence of HDV is 9% while earlier prevalence data are not available from Nigeria [6]. According to the recommendation of the WHO on HIV-ART in adults and adolescents, HBsAg testing should be done before the initiation of ART [24, 25]; however, this is not the rule in most low-income countries including Nigeria.

In this study among HIV-positive individuals in Southwestern Nigeria, 16.1% (n = 50/310) were HBsAg positive, of which 72% (n = 36/50) corresponding to 11.6% overall (n = 36/310) were also positive for HBV DNA. The prevalence of HBsAg observed in this study is high compared to previous reports from Africa, like South Africa with 7.6% [26], 1.1% in Mali [27], and 6.5% in south eastern Nigeria [28]; contrarily it is lower than other reports from southwestern Nigeria with a HBsAg prevalence of 28.4% [29] and 21% in Cameroon [30]. The relatively high HBsAg prevalence observed in this study indicates that HBV is highly endemic in southwestern Nigeria and may possibly be a consequence of increasing drug resistant HBV strains circulating in this region [31].

Of the 50 HBsAg positive individuals, 16.1% (n = 8/50) were positive for HDV RNA. The prevalence of HDV in this study is higher than the reported global HDV prevalence of 5% [32] and a recent report showing 9% HDV prevalence in the general population in Southwestern Nigeria [6]. The high HDV prevalence in our study may be due to the analysed study cohort. Since HIV and HBV share the same transmission routes, co-infection is common and might explain why the HBV and HDV prevalence is higher in this group than in the general population. In this study, only HDV genotype 1 was detected and this agrees with previous reports which show that HDV-1 is ubiquitous and has been previously identified also in southwestern Nigeria [6]. Notably, we found that all HDV RNA positive samples were negative for anti-HDV ELISA which is probably due to the lower sensitivity of the anti-HDV ELISA in comparison to the PCR assay [6].

Liver disease due to HDV is known to progress faster in HIV-positive individuals independent of successful ART [33]. In this study, characteristics such as HBV viral load and CD4 cell count did not significantly differ between the ART-experienced and the ART-naïve cohort. Furthermore, detection of antibodies against the delta antigen did not seem to correspond with molecular detection of HDV RNA. We therefore would recommend that samples tested negative by anti-HDV ELISA, should be retested by molecular methods especially in high risk groups.

In the past decade a number of new treatment options and regimens for HIV infection have been developed pioneering the control of the HIV infection and progression [10]. Nowadays also low-income countries like Nigeria have access to new and highly effective antiretroviral drugs (ARVs) but the old drugs which have a low barrier to resistance are still in use and widely distributed. There are a number of approved ARVs usually used as combination therapy including nucleoside reverse transcriptase inhibitors (NRTI), non-nucleoside reverse transcriptase inhibitors (NNRTI), protease inhibitors (PI), entry inhibitors, and HIV integrase inhibitors (INI). Tenofovir (TDF/TAF) and lamivudine (3TC), both NRTIs are also effective for the control of HBV replication and are recommended for the treatment of chronic HBV infection.

Antiviral drug resistance has been described for HBV and HIV and the selection of HBV DRMs in patients co-infected with HIV under ART with nucleoside analogues have been previously reported [3, 24]. Amino acid (aa) substitutions found in this study include rtL180M, rtV173L, rtM204V/I among individuals on ART however, in one drug-naïve individual, aa substitutions L180M, M204V and S202I have also been detected. This drug-naïve individual might have been infected with a drug resistant HBV strain in circulation or selected the mutation due to factors other than antiviral therapy pressure. Primary aa-substitution rtM204V was the most common in this study, conferring lamivudine-like resistance which has been reported previously to emerge at an annual rate of 15–20% in HIV-1/HBV co-infected individuals in developed countries [24]. Our finding implies that drug resistant HBV-strains are in circulation in Nigeria, which may have originated from ART-patients and may also be transmitted to susceptible individuals. Here, we found a high prevalence of resistance mutations to nucleoside analogues and predominantly mutations usually selected by lamivudine (rtL180M (25%; n = 9/36), rtV173L (22.2%), n = 8/36; rtM204V/I (27.8%; n = 10/36) and rtS202I (2.8%; n = 1/36) (Table 2) in the HIV/HBV co-infected individuals collectively. Additionally, we detected HBV DRMs also in HIV/HBV/HDV co-infected individuals.

The control of HBV infections with antivirals has been reported to avert liver disease progression in most of HIV co-infected patients, however, the emergence of drug resistance and lack of satisfactory treatment options for HDV-infection remains a major threat [34].

It has been recommended that HBV screening should be carried out in HIV patients at baseline before commencement of ART therapy however, our findings demonstrates that not only baseline HBV testing is important in HIV-infected populations but also HBV resistance and HDV testing may be necessary before initiation of ART to ensure a well-adapted and more personalized therapy option.

The limitations to this study are the relatively small sample size and the inability to follow-up the participants. We were also not able to perform additional serological tests due to limited sample volume, e.g. to determine anti-HBc (core) IgM of HBsAg-positive samples to differentiate chronic HBV from acute cases as they may behave differently.

## Conclusions

Coinfections of HIV-positive individuals with Hepatitis B and D viruses are common especially in regions where these virus infections are endemic. Coinfection of HIV with HBV and additionally HDV can be associated with more severe liver disease than mono-infection with HBV. HIV-ART will also affect HBV since HBV DRMs can occur. Accordingly, this study indicates that HBV/HDV coinfections are common in HIV-positive individuals under ART in Nigeria and the prevalence of viremic HDV is high in HIV-positive persons in Nigeria (16.1%). Moreover, a high number of HBV drug resistance mutations were detected underscoring the need for HBV screening prior to starting ART.

## Data Availability

All data generated or analysed during this study are included in this published article.

## Abbreviations

HIV: Human immunodeficiency viruses
HBV: Hepatitis B virus
HDV: Hepatitis D virus (delta virus)
DRM: Drug resistance mutation
ART: Anti-retroviral therapy
TDF/TAF: Tenofovir alafenamid
3TC: Lamivudine
rt: Reverse transcription
